# A demonstration based on multi-omics transcriptome sequencing data revealed disulfidptosis heterogeneity within the tumor microenvironment of esophageal squamous cell carcinoma

**DOI:** 10.1007/s12672-023-00711-5

**Published:** 2023-06-12

**Authors:** Fuxing Liu, Donglan Yuan, Xia Liu, Shichao Zhuo, Xinyun Liu, Haihui Sheng, Min Sha, Jun Ye, Hong Yu

**Affiliations:** 1 Department of Pathology, The Affiliated Taizhou People’s Hospital of Nanjing Medical University, Taizhou, 225300 Jiangsu China; 2 Department of Gynecology, The Affiliated Taizhou People’s Hospital of Nanjing Medical University, Taizhou, 225300 Jiangsu China; 3 Department of Pathology, Xuzhou Central Hospital Affiliated to Nanjing University of Chinese Medicine, Xuzhou, 221000 Jiangsu China; 4 Translational Medicine Center, The Affiliated Taizhou People’s Hospital of Nanjing Medical University, Taizhou, 225300 Jiangsu China

**Keywords:** Disulfidptosis, Esophageal squamous cell carcinoma, Risk score, Tumor microenvironment, CD96

## Abstract

**Background:**

It is of great concern to identify prognostic signatures for the prediction and prediction of esophageal squamous cell carcinoma (ESCC), which is the lethal pathological type of malignancy.

**Method:**

Bulk RNA sequencing and scRNA-seq data were retrieved from GSE53624, GSE53622, and GSE188900. Disulfidptosis-related differentially expressed genes (DEGs) were identified between disulfidptosis-high score and disulfidptosis-low score groups. Functional annotation of DEGs were analyzed by Gene Ontology (GO). Consistent clustering and co-expression modules were analyzed, and then constructed a risk score model via multivariate Cox regression analysis. Immune infiltration and immunotherapy response analyses were conducted based on risk score. qRT-PCR, colony formation assay, and flow cytometry analysis were conducted in KYSE-150 and TE-1 cell lines.

**Results:**

Seven genes (CD96, CXCL13, IL2RG, LY96, TPK1, ACAP1, and SOX17) were selected as marker genes. CD96 and SOX17 are independent prognostic signatures for ESCC patients, with a significant correlation with infiltrated immune cells. ESCC patients had worse response to nivolumab in the high-risk group. Through cellular experiments, we found that CD96 expression was associated with apoptosis and cell cycle ESCC cells.

**Conclusion:**

In a word, the risk score based on disulfidptosis is associated with prognosis and the immune microenvironment, which may direct immunotherapy of ESCC. The key gene of risk score, namely CD96, plays a role in proliferation and apoptosis in ESCC. We offer an insight into the exploration of the genomic etiology of ESCC for its clinical management.

## Introduction

It is of considerable importance to acknowledge that esophageal squamous cell carcinoma (ESCC) is a highly lethal pathological category of malignancy worldwide, which is the sixth reason of death in human cancer [1, 2]. There are more than half of ESCC patients have distal metastases when they are diagnosed, and 5-year survival of ESCC is as low as 20% [3]. Genomic variations in cancer cells are widely deemed as drivers for the progression of ESCC [4]. Therefore, detecting effective genomic signatures is crucial for improving therapeutic effects of ESCC.

Growing evidence illustrates the importance of tumor microenvironments (TME) in ESCC tissues. TME contains vascular endothelial cells, immune infiltrated cells and stromal cells with pro-oncogenic and tumor suppressive effects [5]. Multiple immune cells are often associated with cancer cell metastasis and unfavourable prognostic outcomes of disease. Depleted T cells and NK cells are reported to be the main proliferating cellular components in ESCC. Tumor-associated macrophages can play a tumor-promoting role by inducing immune escape mechanisms [6]. Highly saturated immune cells were strongly associated with the prognosis of ESCC [7]. Recent studies reported the correlation of prognostic markers of ESCC with immune cells [8]. The number of NK cells and macrophages were enormously correlated with post-operative outcomes in ESCC patients [9]. Macrophages affect angiogenesis, tumor cell motility, used for cancer immunotherapy. Macrophages may be polarized into tumor suppressive M1 or pro-oncogenic M2 phenotype in TME [10]. Furthermore, it was reported that up-regulation of FOXO1 in tumor tissues leads to M0 macrophage polarization and M2 macrophage infiltration into TME, leading to poor prognosis in ESCC patients [11]. Overall, an in-depth understanding of TME status in ESCC patients helps to elucidate their genomic profile and improve prognosis.

While treatment of ESCC has improved, the characteristics of high recurrence, metastasis, and resistance consistently lead to unsatisfactory prognoses [12]. Primary ESCC treatments include esophagectomy, chemotherapy, and radiotherapy. Esophagectomy, surgical removal of the esophagus, can be curative but has high morbidity [13]. Limitations include recurrence, metastasis, and treatment resistance. Chemotherapy and radiotherapy may fail to eradicate all tumor cells, leading to disease persistence, resistance, and metastasis. Immunotherapy is an innovative therapeutic approach with remarkable therapeutic effects in advanced cancers [14, 15]. The burgeoning interest in immunotherapy for ESCC is aimed at enhancing patient survival outcomes, further emphasizing its significance in comprehensive treatment. Recently, the treatment of nivolumab exhibited a good survival in patients with ESCC [16]. Therefore, it is an urgency to find novel biomarkers to accurately predict the outcome of ESCC patients receiving immunotherapy.

In the study’s aims, we explore the clinical implications of identifying novel prognostic marker genes, highlighting their potential to influence patient stratification, inform personalized treatment strategies, and improve overall outcomes in ESCC management. Diverse immune infiltrating cells combined with marker genes were analyzed in different risk groups, aiming to assess the stable value of the prognostic model.

## Methods

### Data collection and processing

The comprehensive RNA-seq expression profiles of 177 ESCC patients, accompanied by pertinent clinical data from GSE53622 and GSE53624 datasets, were meticulously procured from the Gene Expression Omnibus (GEO) database. Additionally, ESCC single-cell RNA-seq (scRNA-seq) data were obtained from the GSE188900 dataset, encompassing a sample size of four (N = 4).

Unsupervised clustering of the cells is analyzed using the Seurat package and visualized using t-distributed stochastic neighbor embedding (tSNE). The t-SNE algorithm is a nonlinear dimensionality reduction technique. Seven cell populations, including B cells, T cells, epithelial cells, mast cells, myeloid cells, fibroblast cells, and endothelial cells, were manually gated with cell-specific expression markers from 4 ESCC samples. AddModuleScore function scored the gene set enrichment of disulfidptosis pathway to obtain “Disulfidptosis score”. Each immune cell population was divided into two groups: disulfidptosis-high score and disulfidptosis-low score. The PROGENy score was calculated to show the scores of tumor-related pathways in different cell populations.

### Identification of differentially expressed genes

The FindAllMarkers function, an integral component of the Seurat R package, was employed to adeptly discern differentially expressed genes (DEGs) between the high-score and low-score cohorts, facilitating a thorough comparative analysis. The adjusted *P* < 0.05 and |log_2_fold change(FC)| > 0.585 are statistical significance.

### Function annotation analysis

Gene Ontology (GO) analysis of DEGs were performed using clusterProfiler R package (version 4.0.5) between Disulfidptosis score-high and Disulfidptosis score-low groups. False discovery rate (FDR) cutoff of 0.05 was used for functional categories with significantly enriched functional content.

### Unsupervised cluster analysis

ConsensusClusterPlus R package was employed for consistent clustering analysis on ESCC samples. Clustering analysis by K-means algorithm is an iterative process. The optimal value of K was determined using the elbow method, and the identified clusters informed our study’s findings, providing insight into the molecular subtypes and their prognostic relevance in ESCC. The classification was repeated 500 times to ensure stability. Cumulative distribution function (CDF) curve ascertained the optimal clustering number. Principal component analysis (PCA) was carried out for examining the heterogeneity in different clusters.

### Weighted gene coexpression network analysis (WGCNA)

WGCNA was used to eliminate the offset and obtain disease-related modules for further analysis. The WGCNA analysis in our study involved determining the correlation coefficient, enabling the identification of key modules and hub genes, which facilitated a deeper understanding of the underlying molecular mechanisms in ESCC. WGCNA analysis was carried out using WGCNA R package as per expression profiles and clinical features of ESCC samples. A correlation coefficient was calculated between clinical features and modules in order to identify key modules. The hub genes of modules were confirmed according to the most correlated clustering.

### Construction of prognostic risk model

Multivariable cox regression was analyzed for DEGs by tinyarray R package. Prognostic marker genes were screened at the threshold of P < 0.05 in forest map. Construction of a prognostic model was performed on the marker genes from GSE53624 dataset and GSE53622 dataset for training and external validation. Criteria for high-score and low-score groups in disulfidptosis were determined using a predetermined threshold, the median value of risk score was applied as the optimal threshold point for distinguishing low-risk group and high-risk group. Kaplan–Meier survival analysis with log-rank test was evaluated the predictive value of the model. The protein expression levels and the correlations between marker genes were compared in groups and visualized via heatmap, box plot, and pie chart.

### Immune infiltration analysis

The TME of ESCC tissues was analyzed using xCell algorithms, which was applied for calculating the cellular abundance of 67 types of immune cells on TME. Immune infiltration was visualized using heatmap, box plot, and scatter plot.

### Immunotherapy response

Checkmate-577 involved 794 participants with resected stage II/III ESCC, testing nivolumab as adjuvant therapy. Patients with residual tumor or autoimmune disease were excluded. According to Checkmate-577, nivolumab, a fully human IgG4 PD-1 immune checkpoint inhibitor antibody, is the most effective adjuvant treatment for non-metastatic ESCC with residual disease. The performance of risk score was assessed using a separate cohort from the Checkmate-577 study. All subjects were evaluated by the response to therapy. They were divided into response group (CR) and non-response (non-CR). The difference of proportions of response condition were compared between high-risk and low-risk groups.

### Cell culture and transfection

In order to identify the potential biological function of candidate genes, we conducted series of cellular functional experiments. Human esophageal cancer KYSE-150 and TE-1 cell lines were purchased from the Type Culture Collection of the Chinese Academy of Sciences, Shanghai, China. They were cultured with RPMI-1640 medium (Gibco, USA) supplemented with 10% of fetal bovine serum (Gibco, USA) at 37 °C in 5% CO_2_ atmosphere.

The small interfering (si) RNA (si-CD96) was constructed for knockdown the CD96, and transfected into both cell lines using Lipofectamine 3000 (Thermo Fisher, USA).

### Colony formation assay

After digested cells in the logarithmic growth phase, the cells were re-suspended into cell suspension in the medium then counted. Each experimental group was inoculated with 500 cells/well in 6-well plate. The culture was continued until 14 days or the number of cells in most single clones was greater than 50. During the process, the medium was changed every 3 days and the cell status was observed. After the experiment was completed, the 6-well plate was washed with PBS, and 1 ml 4% paraformaldehyde was added to each well and fixed for 60 min. After that, the cells were washed with PBS, and 1 ml crystal violet dye was added to each well. After 20 min, the cells were washed with PBS for several times. Finally, the 6-well plate was dried and photographed.

### Cell cycle and apoptosis assays

Logarithmic cells were inoculated into 6-well plates (2 × 10^5^ cells/well), where each well was cultured with 2 ml culture medium and transfected. After transfection, trypsin was used to digest the cells and cells were gently blow to avoid cell damage and cell adhesion. The supernatant was centrifuged (1500 rpm for 4 min) and the cells were washed with pre-cooled PBS for 2–3 times. A small amount of PBS was added to the cell precipitate and the cells were gently and fully re-suspended. Then the suspended cells were added to 70% icy ethanol precooled at 4 ℃ for fixation, then gently blown, sealed with a sealing film, and kept at 4 ℃ overnight. After that, the cells were centrifuged for 4 min (2000 rpm) and the supernatant was removed. The cells were washed twice with PBS to remove the supernatant. 100 µl 100 µg/ml RNase A and 0.2% Triton X-100 were added to suspend cells and placed in 37 ℃ water for 30 min. 400 µl 50 µg/ml PI was added and mixed with vortex oscillation, incubated at room temperature and away from light for 30 min. The cell cycle was measured by flow cytometry, and then the cell cycle phase distribution was analyzed.

For apoptosis assays, the cells were cultured and collected in the same way as in the cell cycle experiments. The collected cells were washed with PBS, the supernatant was abandoned after centrifugation, and the cells were re-suspended with 500 µl diluted 1×Annexin V Binding Buffer working solution, and then with 5 µl Annexin V-APC and 5 µl 7-AAD staining solution. After mixing with gentle vortices, incubate at room temperature and away from light for 20 min, and test on the machine immediately after the reaction is complete.

### Quantitative real-time PCR (qRT-PCR)

Total RNA was extracted from TE-1 and KYSE-150 cells by Trizol reagent. After cDNA synthesis using the NovoScript All-in-One SuperMix (Novoprotein, China), qRT-PCR for mRNA was performed with Step One Plus Real-Time PCR system (Applied Biosystems, USA) in consjunction with Hieff qPCR SYBR Green Master Mix (Novoprotein, China) for the real-time PCR reaction. The qPCR protocol was set of 95 °C for 5 min, 40 cycles at 95 °C for 10 s, and 60 °C for 60 s. GAPDH was used as an internal standard control. Data were calculated by comparing Ct values for each sample with three replications.

### Statistical analysis

All statistical analysis was carried out through R software. Cox regression analysis was performed by survival and survminer R package. *P* < 0.05 was deemed as significance.

## Results

### ScRNA-seq analysis of ESCC samples

**Fig. 1:**
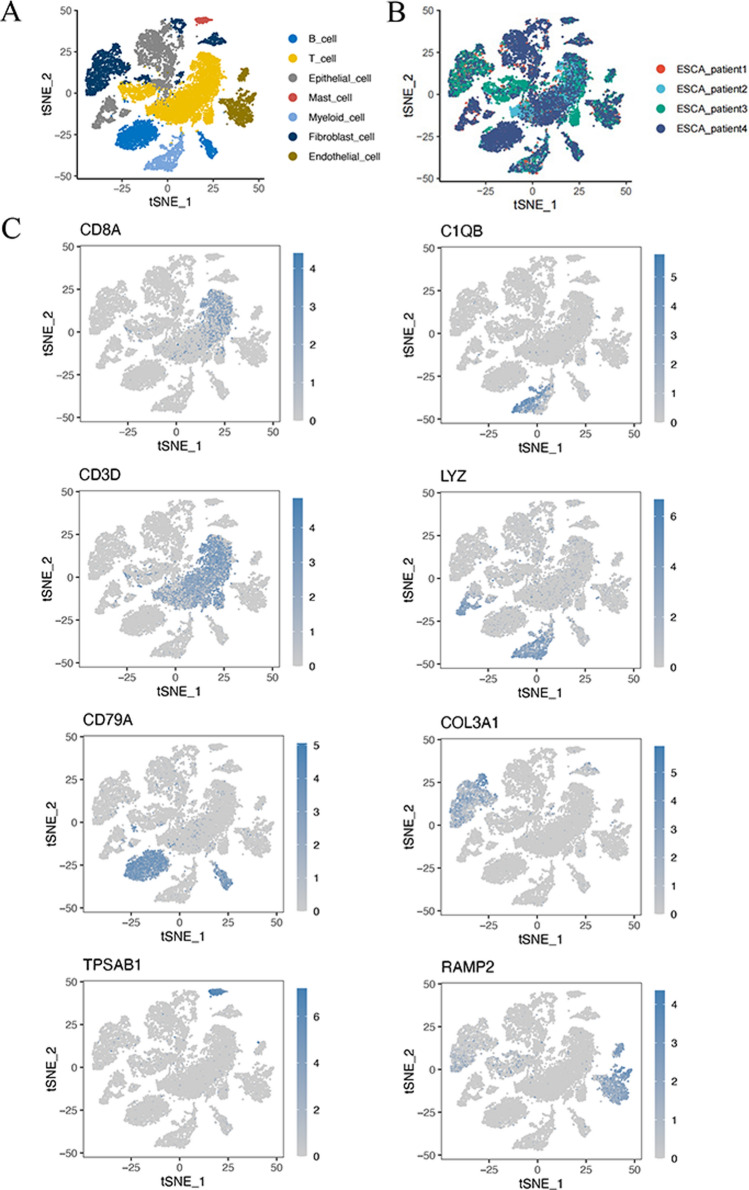
Subtype identification results of ESCC cohort. **A** tSNE plot of 7 cell populations after dimension reduction. **B** tSNE plot of cell distribution in 4 patients with ESCC. **C** tSNE map of cluster marker genes in different cell populations

**Fig. 2:**
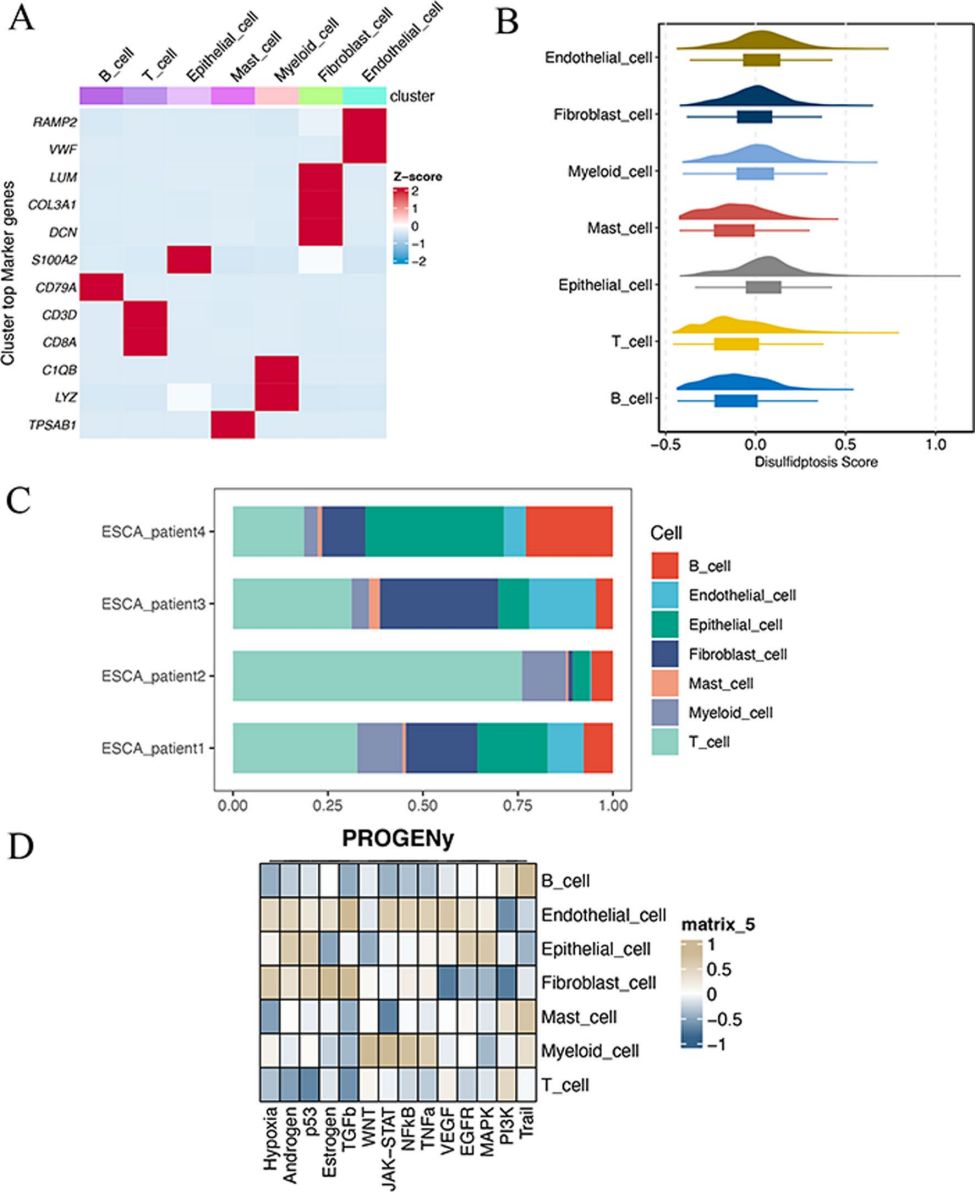
**A** Dotplot of cluster top 12 marker genes in 7 cell populations. **B** Ridge plot of 7 cell populations based on disulfidptosis score. **C** Accumulation diagram of the proportion of different cell subgroups in 4 patients with ESCC. **D** Progeny scores of 7 cell populations

The distributions of B cells, T cells, epithelial cells, mast cells, myeloid cells, fibroblast cells, and endothelial cells in 4 ESCC patients are shown in Fig. 1A and B. High expression of TPSAB1, RAMP2, COL3A1, CD79A, CD3D, CD8A, C1QB, and LYZ mostly contributed to different clustering of 7 cell populations (Fig. 1C). The correlation between top 12 marker genes and 7 cell subgroups was analyzed in Fig. 2A. The Disulfidptosis scores of each cell subgroups were exhibited in Fig. 2B, which quantifies the level of oxidative stress. Figure 2C exhibited the proportions of different cell populations in 4 patients from GSE188900 dataset. The PROGENy result showed the relationship between different cell populations and tumor-related pathways in Fig. 2D, which higher PROGENy scores indicate stronger pathway activity.

### Identification of DEGs and function enrichment analysis

**Fig. 3:**
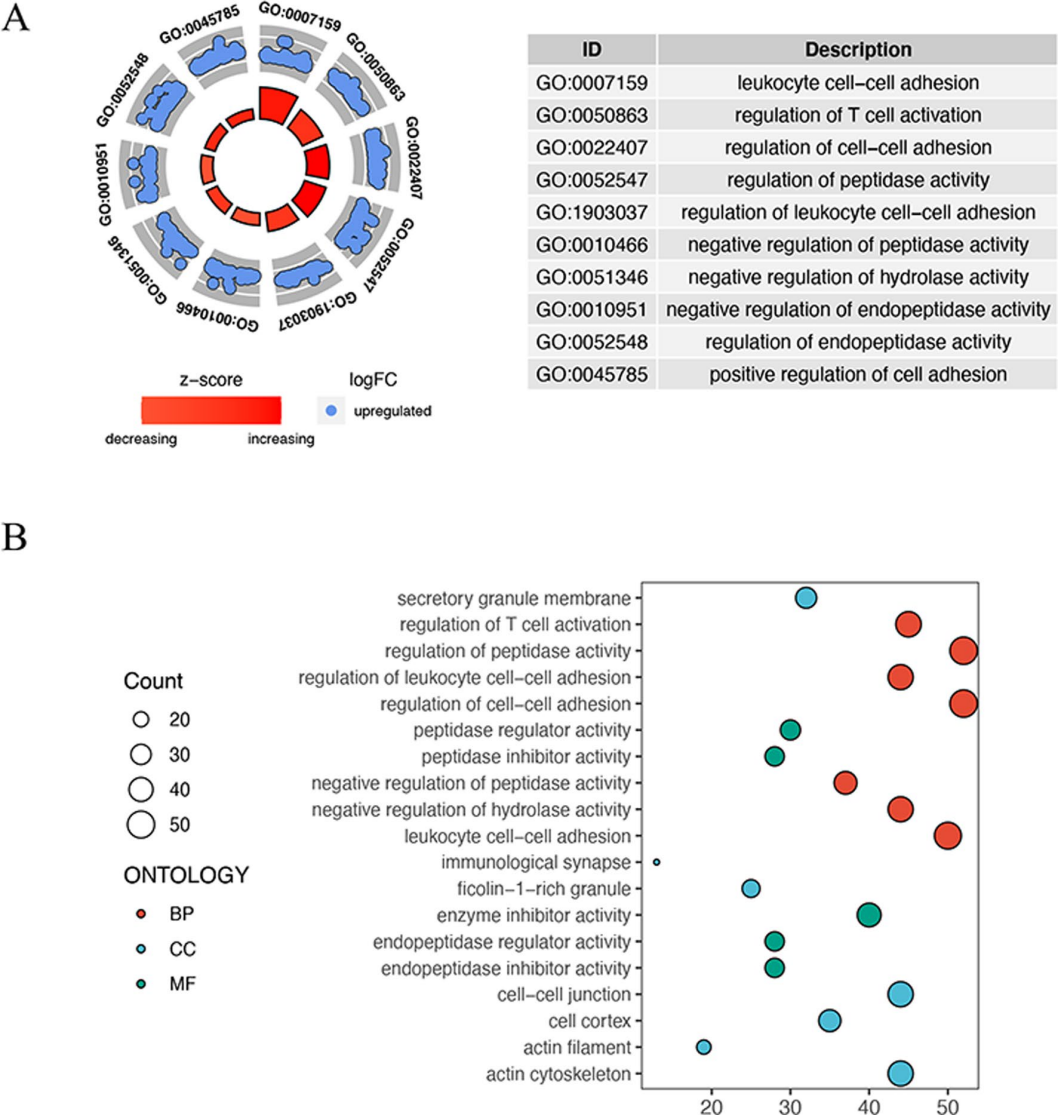
Functional enrichment analysis of 443 DEGs. **A** Top GO terms of DEGs. **B** GO enrichment terms of DEGs for BP (biological process), CC (cellular component), and MF (molecular function)

Based on disulfidptosis score grouping, totally 443 DEGs were identified at the threshold of adjusted P < 0.05 and |log_2_FC| > 0.585 between high-score and low-score groups. Top 10 GO terms were shown in Fig. 3A. GO analysis results of molecular function showed that these DEGs are enriched in leukocyte cell-cell adhesion and the positive regulation of T cell activation, peptidase activity, leukocyte cell-cell adhesion, and cell-cell adhesion, as well as the negative regulation of hydrolase activity (Fig. 3B).

### Consistent clustering

**Fig. 4:**
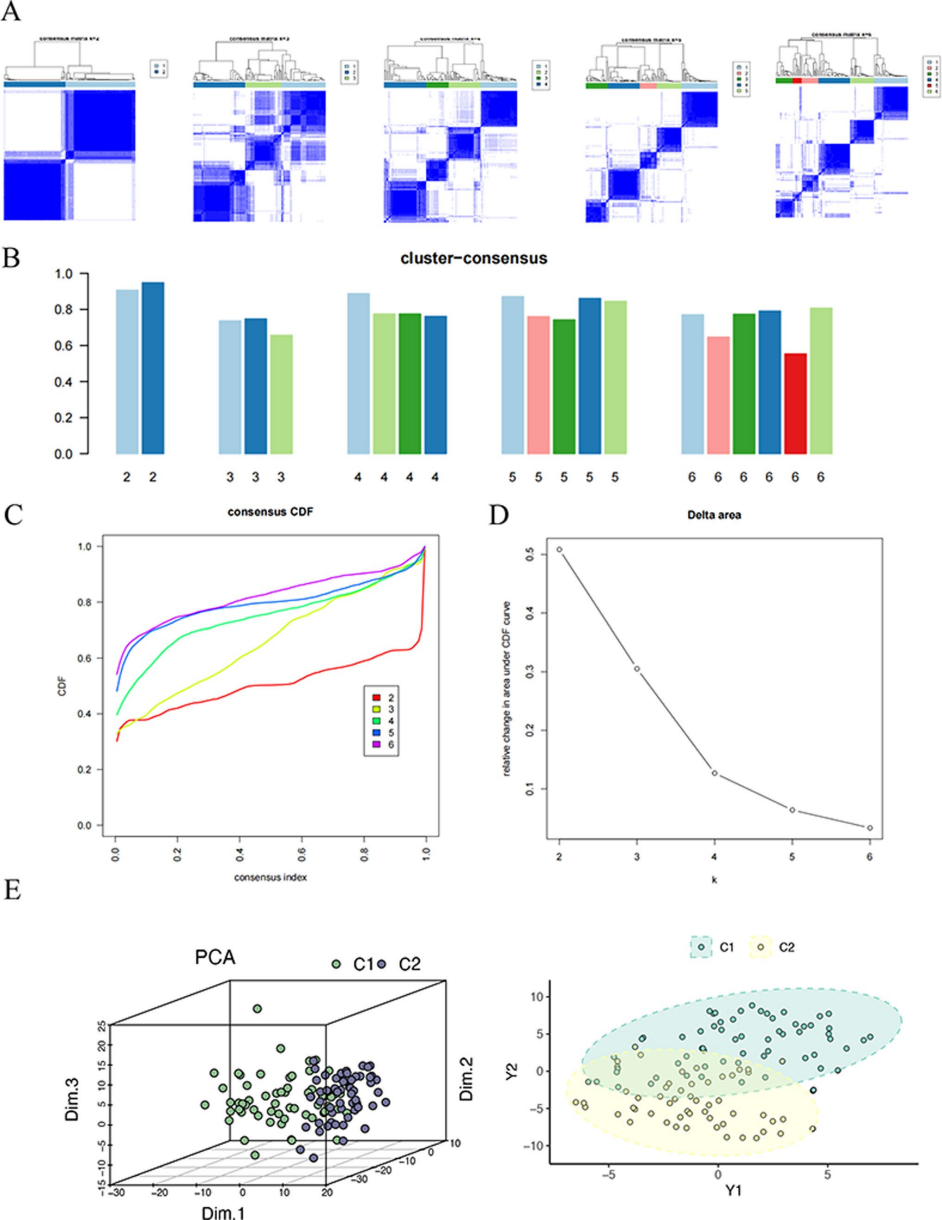
Consensus matrix plot of clustering in ESCC dataset. **A** Unsupervised cluster plots of distinct groups (k = 2 to 6). **B** The cluster-consensus plot of the different classification numbers (k = 2 to 6). **C** CDF curve of consistent clustering. **D** Relative change in area under the CDF curve of consistent clustering. **E** PCA analysis of ESCC patients in cluster1 and cluster2

The most optimal clustering effect was achieved when K = 2, as depicted in Fig. 4A and B. The cumulative distribution function (CDF) curve of the clustering is illustrated in Fig. 4C, signifying the function’s progression as k assumes varying values. Figure 4D shows the change of the area under the CDF curve when k was relative to k-1. The results of PCA analysis showed that the scattered distributions of the patients in the cluster2 (C2) and the cluster1 (C1) from the ESCC dataset had two-way features (Fig. 4E), suggesting the prognostic potency of different clusters.

### Construction of co-expression module by WCGNA

**Fig. 5:**
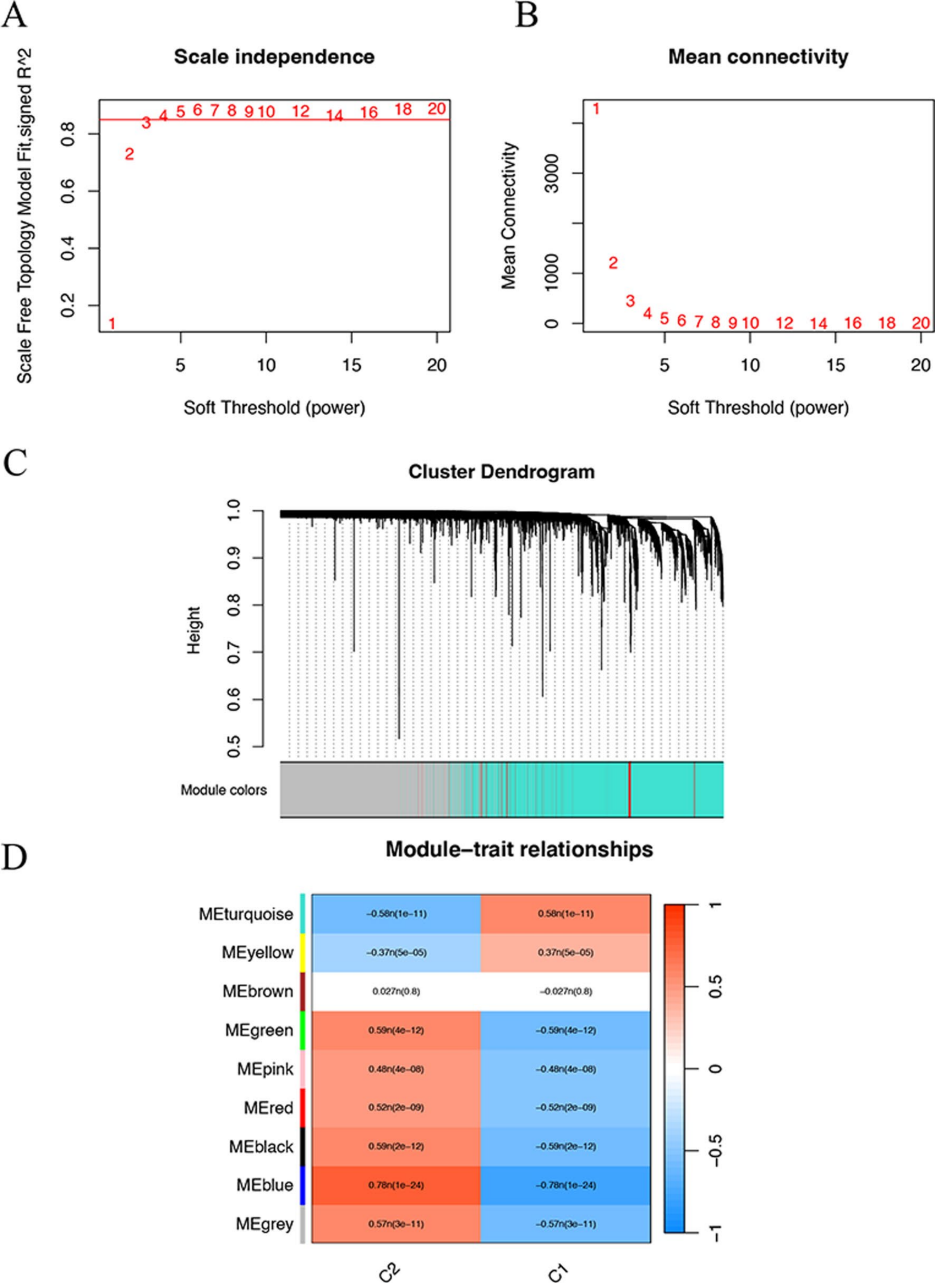
WCGNA network and co-expression module based on two consistent clusterings. **A** Scatter map of soft threshold distribution. **B** The mean connectivity for soft threshold powers. **C** Clustering tree graph of genes in the modules. Each branch represents one gene, and every color below represents one co-expression module. **D** Heatmap of module-trait relationships between key modules and two clusterings

After testing thresholding powers from 1 to 20, a power value of 4 was selected (correlation coefficient is more than 0.8) in Fig. 5A and B, leading to the generation of 9 modules (Fig. 5C). In the module clustering, blue and turquoise module are most significant modules, which are closely related with C1 and C2 (Fig. 5C). The blue module was the most positive module correlated with 117 ESCC patients in C2, and the turquoise module was the most negative module correlated with 117 ESCC patients in C2 than were those of the other modules (Fig. 5D). Consequently, genes encompassed within the blue module may be implicated in the tumorigenesis of ESCC, while those residing in the turquoise module may exhibit tumor-suppressive properties.

### Construction of prognostic model

Seven genes (CD96, CXCL13, IL2RG, LY96, TPK1, ACAP1, and SOX17) were subsequently selected from the module genes via multivariate Cox regression analysis to establish a prognostic model. Calculation formula of risk score as follows: Risk score = [CD96 × 0.399] − [CXCL13 × 0.030] − [IL2RG × 0.178] − [LY96 × 0.238] − [TPK1 × 0.348] − [ACAP1 × 0.277)] − [SOX17 × 0.538]. All ESCC objects were classified into high-risk and low‐risk groups in the light of median risk scores.

**Fig. 6:**
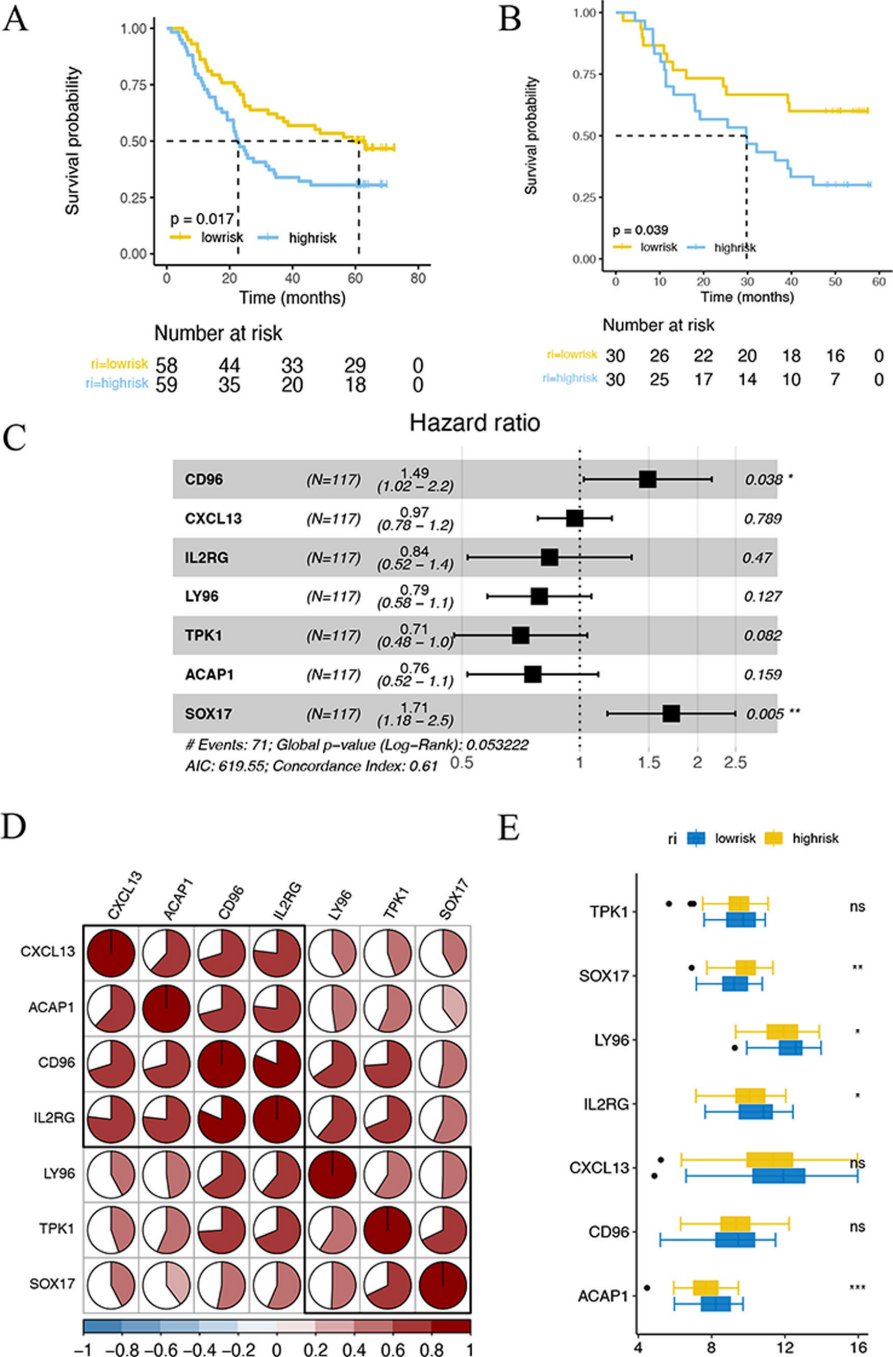
Identification of prognostic marker genes in ESCC patients. **A** Kaplan–Meier curve of risk prognostic model of ESCC patients in training set. **B** Kaplan–Meier curve of risk prognostic model of ESCC patients in validation set. **C** Forest map of multivariate cox correlation analysis of OS-related marker genes in patients with ESCC. **D** Corrplot of the correlation of 7 marker genes. **E** Boxplot of expression levels of 7 marker gene between high-risk and low-risk groups

Kaplan–Meier curve (Fig. 6A and B) exhibited high-risk patients had remarkably worse OS than low-risk patients in training and validation sets (P < 0.05). The multivariable Cox regression revealed significant variations of marker genes, suggesting CD96 and SOX17 are independent prognostic signatures for ESCC patients with great significance (Fig. 6C). Correlation analysis among 7 marker genes was conducted (Fig. 6D). As shown in Fig. 6E, SOX17, LY96, IL2RG, and ACAP1 protein expression levels are remarkably different between risk groups (P < 0.05).

### Immune infiltration landscape

**Fig. 7:**
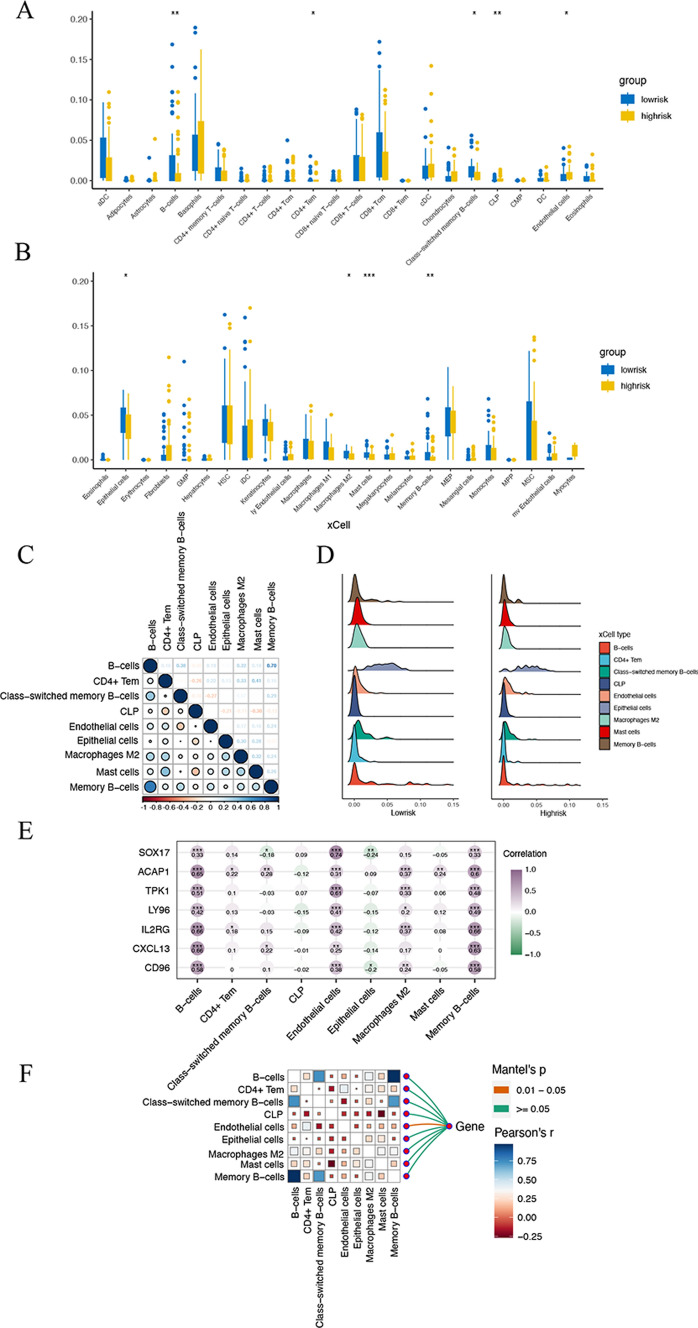
The immune infiltration in the high and low risk groups of ESCC patients. **A**, **B** Boxplot of immune infiltration between high-risk and low-risk groups based on xCell algorithm. **C** Corrplot of 9 immune cells. **D** Ridge plot of 9 immune cells between high-risk and low-risk groups. **E** Heatmap of the correlation between 7 marker gene expression and the proportion of 9 immune infiltrating cells. **F** Corrplot of correlations of 9 immune cells and genes

The proportions of immune cells in ESCC calculated by xCell algorithm are shown in Fig. 7A and B. Epithelial cells, macrophages M2, mast cells, memory B cells, B cells, CD4+ Tem cells, class-switched memory B cells, CLP, and endothelial cells showed noteworthy difference between high-risk and low-risk groups (P < 0.05). Correlations among all 13 immune cells as well as are shown in Fig. 7C. The positive correlation categories comprised memory B cells and B cells, and mast cells and CD4+ Tem cells, with coefficient more than 0.4. The negative correlation categories comprised CLP and CD4+ Tem cells, endothelial cells and class-switched memory B cells, CLP and mast cells, and CLP and epithelial cells, with coefficient less than − 0.2. The proportions of epithelial cells, macrophages M2, mast cells, memory B cells, B cells, CD4+ Tem cells, class-switched memory B cells, CLP, and endothelial cells evaluated by xCell algorithm in low-risk group are different from those in high-risk group in Fig. 7D. Associations of 7 prognostic marker genes and 9 infiltrated immune cells was exhibited in Fig. 7E, F.

**Fig. 8:**
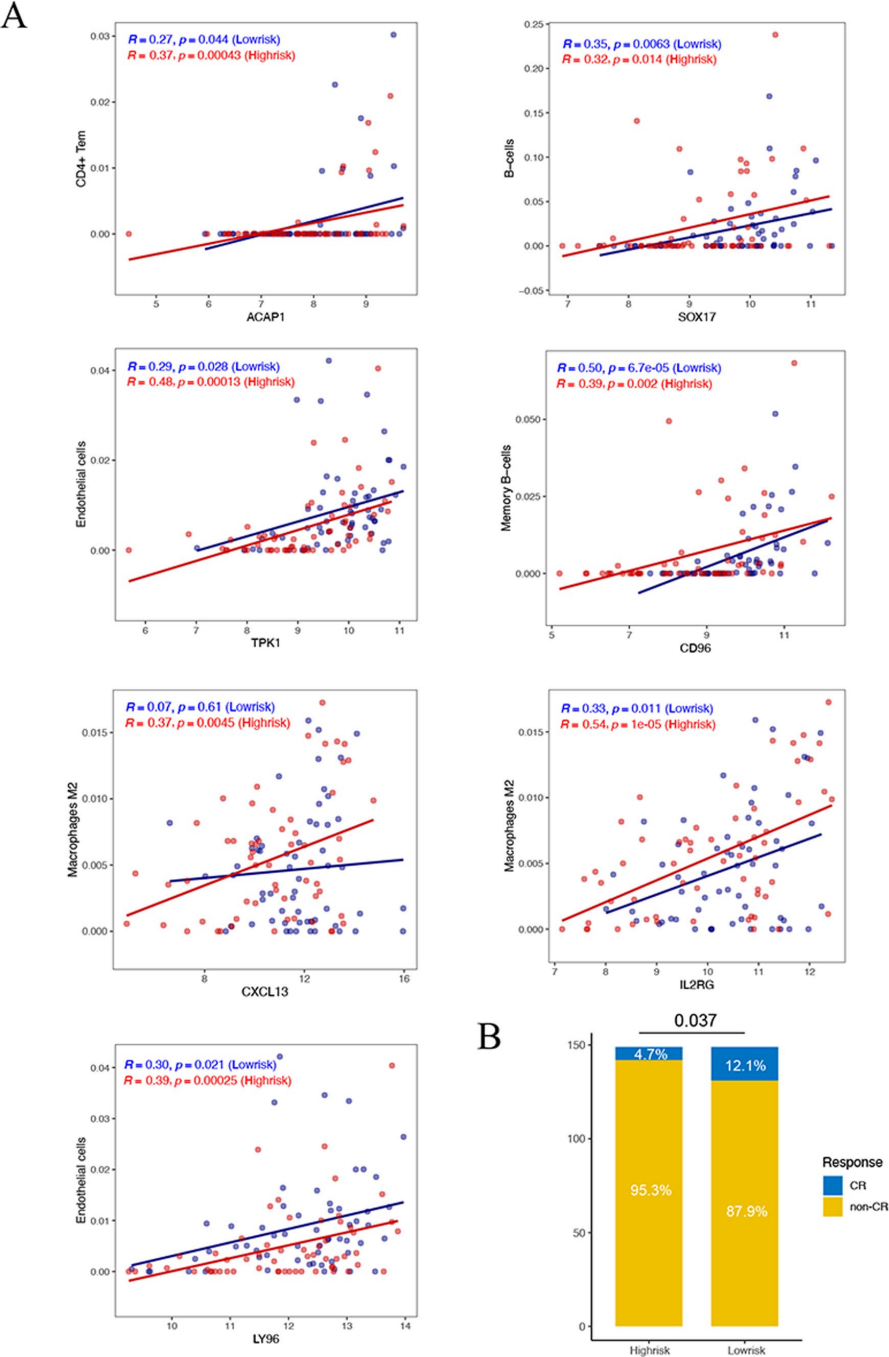
**A** Scatter plot of the correlation coefficient between marker genes and immune cells in high-risk and low-risk groups. **B** Histogram of CR and non-CR between high-risk and low-risk groups in checkmate-577 queue

As shown in Fig. 8A, ACAP1 and SOX17 have significant positive correlations with infiltrated CD4+ T cells and B cells in both groups, respectively. TPK1 and LY96 are positively associated with endothelial cells in high-risk and low-risk groups (P < 0.05). Both IL2RG and CXCL13 have greatly positive correlations with macrophages M2 cells in high-risk groups (P < 0.005), while IL2RG is positive correlated with macrophages M2 cells in low-risk groups with great significance (P = 0.011). CD96 is vastly positively associated with memory B cells between risk groups (P < 0.005).

### Immunotherapy analysis

The findings illustrated in Fig. 8B demonstrated that patients with ESCC in the high-risk group exhibited a notably poorer response to PD-1 inhibitors, with 95.3% failing to achieve complete remission (CR), highlighting the potential utility of identified marker genes in the context of immunotherapy.

### Knockdown of CD96 in ESCC cells

**Fig. 9:**
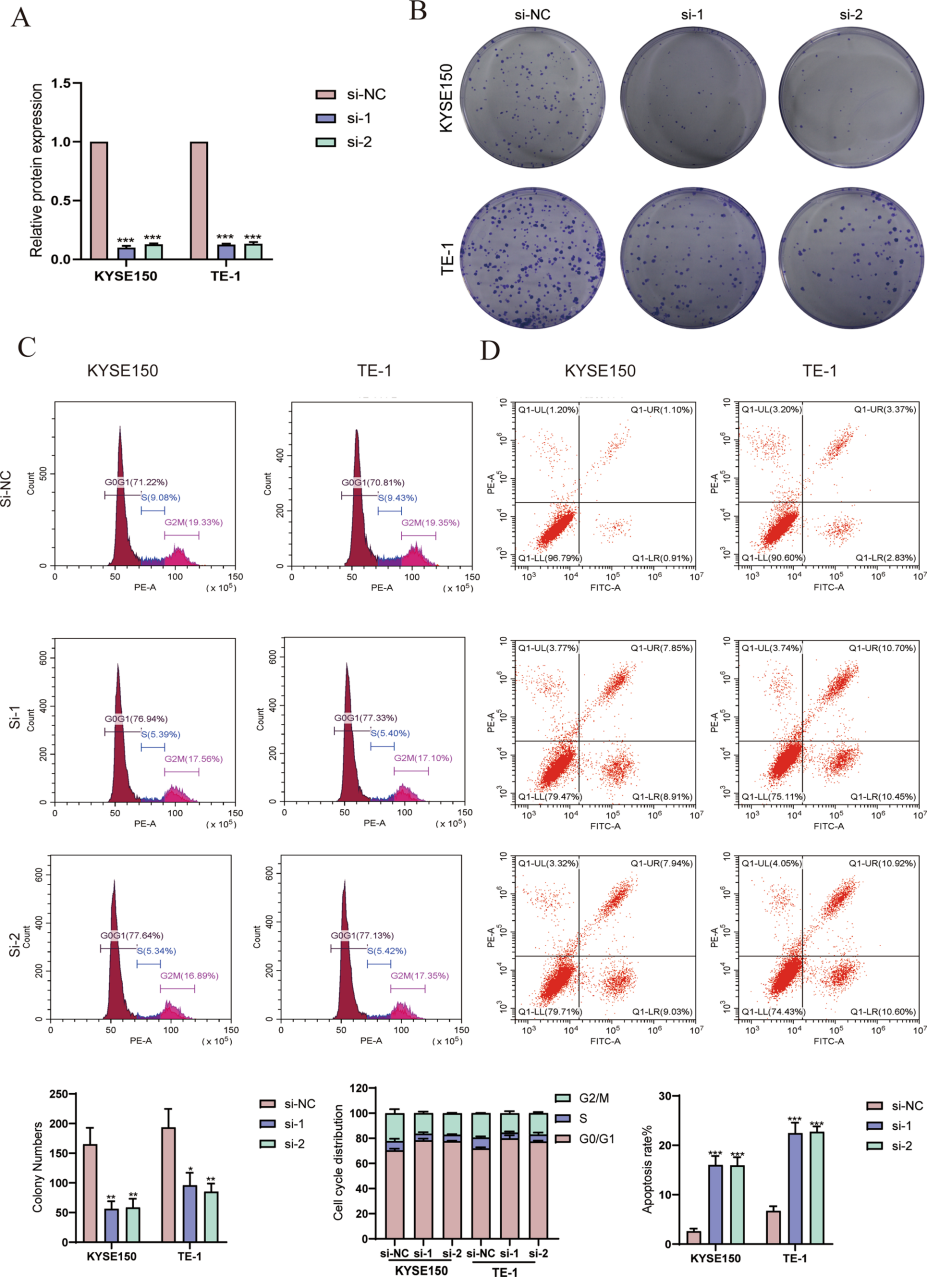
The impact of knockdown of CD96 in cellular growth behaviors in vitro. **A** Knockdown of CD96 expression in ESCC cells revealed by qRT-PCR. **B** The colony formation ability of KYSE-150 and TE-1 cells transfected by si-NC and si-CD96. **C** Cell cycle changes of KYSE-150 and TE-1 cells transfected by si-NC and si-CD96. **D** Cell apoptosis of KYSE-150 and TE-1 cells transfected by si-NC and si-CD96

To better illustrate the effects of risk groups on ESCC cells, we chose CD96 to further study. CD96 mRNA levels in KYSE-150 and TE-1 cells was significantly down-regulated after transfected si-CD96 (Fig. 9A). In Fig. 9B, down-regulation of CD96 significantly attenuated the cell proliferative capacity of ESCC cells. Through flow cytometry analysis, knockdown of CD96 induced cell cycle distribution alterations of KYSE-150 and TE-1 cells, suggesting an incremental proportion of G0/G1 cells and a lessened ratio of S and G2/M cells (Fig. 9C). In addition, knockdown of CD96 was broadly associated with cell apoptosis in both ESCC cell lines (Fig. 9D).

## Discussion

ESCC originates from esophageal squamous epithelial cells with poor prognosis and high mortality rate [17]. Patients with ESCC are likely to suffer from dysphagia because of their exceptional tumor location. It is constructive to find early signatures with high predictive and prognostic value of ESCC. Previous study found that gene expression variations in ESCC objects can mediate biological behaviors of tumor cells and thus influence the course of disease [18]. However, the potential biological functions of ESCC-related key genes are unclear. It is an impressive demand to determine novel valid prognostic signatures in the clinical therapy of ESCC.

Disulfidptosis is a recently characterized cell death modality, involving the disruption of disulfide bonds in proteins. Disulfidptosis is induced by disulfide stress due to the inability of NADPH supply to satisfy the reduction of cystine to cysteine. Overconsumption of NADPH in cells induces actin cytoskeletal protein disulfide cross-linking and cytoskeletal contraction, and ultimately disulfide death [19]. In cancer cells, aberrant disulfidptosis regulation may contribute to tumor progression and resistance [20]. Exploiting this pathway could unveil innovative therapeutic strategies for ESCC, potentially enhancing the efficacy of current treatments and overcoming resistance mechanisms. The identification of cell death mechanisms not only promote the understanding of cell homeostasis, but also provide important ideas for the treatment of tumor diseases. In this study, we evaluated cell populations enriched in disulfidptosis pathway from ESCC samples based on scRNA-seq data and screened disulfidptosis-related DEGs between high-disulfidptosis score and low-disulfidptosis score groups. It seems first study to investigate disulfidptosis-related genes in ESCC, providing evidence for the treatment of the disease and comprehend the biological process of novel cell death in the development of ESCC. In order to excavate potential genes with high prognostic value in ESCC, we performed consistent clustering and co-expression modules, and constructed a prognostic model for prediction. The elucidated marker genes hold potential for directing tailored immunotherapeutic strategies, thereby facilitating the identification of ESCC patients with a higher likelihood of deriving therapeutic benefits. Moreover, these genetic markers may inform the selection of complementary adjuvant treatments, ultimately enhancing treatment efficacy in high-risk ESCC cohorts. Totally 7 potential prognostic biomarkers (CD96, CXCL13, IL2RG, LY96, TPK1, ACAP1, and SOX17) in ESCC were confirmed via multiple bioinformatics analyses. The results showed CD96 and SOX17 are independent prognostic signatures for ESCC patients. Previous studies demonstrated the poor prognosis of low SOX17 protein expression in ESCC patients, and up- regulation of SOX17 in ESCC cells led to reduced lesion formation, decreased cell activity, and metastasis [21]. Increasing findings showed that CD96 served for the diagnosis and treatment of patients with skin cutaneous melanoma [22], glioma [23], and cervical cancer [24]. In the validation part of this study, we confirmed the impact of CD96 down-regulated expression on KYSE-150 and TE-1 cells in cell cycle, reduced proliferation, and increased apoptosis. Knockdown of CD96 is significantly associated with the cellular life activities in ESCC cells, which strongly suggested that CD96 may be a candidate prognostic and therapeutic target of ESCC.

The immune infiltration landscape findings in ESCC unveil disparate patterns of immune cell engagement, indicative of heterogeneous tumor microenvironments. The observed correlations between marker genes and immune cell infiltrates bear considerable clinical relevance, furnishing insights into innovative therapeutic approaches that selectively target distinct immune constituents to enhance patient outcomes.

Recently, immunotherapy was considered as a highly effective and promising treatment strategy due to its good performance in various cancers [25]. Immunotherapy, specifically the utilization of immune checkpoint inhibitors such as PD-1/PD-L1, presents an auspicious avenue for ESCC treatment by amplifying anti-tumor immune responses. Nevertheless, existing challenges encompass inconsistent patient responses and the possibility of immune-related adverse events, thus underscoring the need for continued investigation and refinement [26]. With regard to ESCC, the selective PD-1 inhibitor nivolumab can improve survival in the treatment (Checkmate-577), showing promising activity in early clinical trials of patients with ESCC [27]. However, the effectiveness of these drugs has not always been satisfactory. The innovation of this study is exploring new disulfidptosis-related marker genes to improve outcomes in ESCC patients who are sensitive to nivolumab.

The study has several limitations. First of all, inherent limitations include small sample sizes in training and validation cohorts, potentially affecting generalizability and robustness of findings. Secondly, although bioinformatic methods and cell experiments have verified the reliability of the results, it is still necessary to detect multiple immune cells in tumor samples to verify the conclusion in the future. Lastly, it is imperative to underscore the necessity for in vivo animal models and clinical trials to authenticate the prognostic and therapeutic potential of the ascertained biomarkers, thereby accentuating the significance of further validation investigations within the realm of ESCC treatment. These constraints warrant cautious interpretation and highlight the need for larger, diverse samples in future research to corroborate our results and bolster their applicability to broader clinical contexts.

## Conclusion

In a word, these findings reveal the risk score based on disulfidptosis is associated with prognosis and the immune microenvironment, which may direct immunotherapy of ESCC. The key gene of risk score, namely CD96, plays a role in proliferation and apoptosis in ESCC. We offer an insight into the exploration of the genomic etiology of ESCC for its clinical management. These insights hold potential to shape future research and clinical practice by informing novel therapeutic strategies and refining diagnostic approaches. Ultimately, the findings may lead to improved patient outcomes and enhanced disease management.

## Data Availability

The raw data supporting the conclusions of this article can be obtained from the author, who will hold nothing back.
